# Construction of the High-Density Genetic Linkage Map and Chromosome Map of Large Yellow Croaker (*Larimichthys crocea*)

**DOI:** 10.3390/ijms161125951

**Published:** 2015-11-03

**Authors:** Jingqun Ao, Jia Li, Xinxin You, Yinnan Mu, Yang Ding, Kaiqiong Mao, Chao Bian, Pengfei Mu, Qiong Shi, Xinhua Chen

**Affiliations:** 1 Key Laboratory of Marine Biogenetic Resources, Third Institute of Oceanography, State Oceanic Administration, Xiamen 361005, China; ajingqun@tio.org.cn (J.A.); muyinnan@tio.org.cn (Y.M.); dingyang@stu.xmu.edu.cn (Y.D.); mkqevelyn@stu.xmu.edu.cn (K.M.); mupengfei@stu.xmu.edu.cn (P.M.); 2 Fujian Collaborative Innovation Center for Exploitation and Utilization of Marine Biological Resources, Key Laboratory of Marine Genetic Resources of Fujian Province, Xiamen 361005, China; 3 Shenzhen Key Lab of Marine Genomics, Guangdong Provincial Key Lab of Molecular Breeding in Marine Economic Animals, BGI, Shenzhen 518083, China; lijia1@genomics.cn (J.L.); youxinxin@genomics.cn (X.Y.); bianchao@genomics.cn (C.B.)

**Keywords:** SNP, genetic linkage map, chromosome map, immunity-related genes, hypoxia-related genes, large yellow croaker (*Larimichthys crocea*)

## Abstract

High-density genetic maps are essential for genome assembly, comparative genomic analysis and fine mapping of complex traits. In this study, 31,191 single nucleotide polymorphisms (SNPs) evenly distributed across the large yellow croaker (*Larimichthys crocea*) genome were identified using restriction-site associated DNA sequencing (RAD-seq). Among them, 10,150 high-confidence SNPs were assigned to 24 consensus linkage groups (LGs). The total length of the genetic linkage map was 5451.3 cM with an average distance of 0.54 cM between loci. This represents the densest genetic map currently reported for large yellow croaker. Using 2889 SNPs to target specific scaffolds, we assigned 533 scaffolds, comprising 421.44 Mb (62.04%) of the large yellow croaker assembled sequence, to the 24 linkage groups. The mapped assembly scaffolds in large yellow croaker were used for genome synteny analyses against the stickleback (*Gasterosteus aculeatus*) and medaka (*Oryzias latipes*). Greater synteny was observed between large yellow croaker and stickleback. This supports the hypothesis that large yellow croaker is more closely related to stickleback than to medaka. Moreover, 1274 immunity-related genes and 195 hypoxia-related genes were mapped to the 24 chromosomes of large yellow croaker. The integration of the high-resolution genetic map and the assembled sequence provides a valuable resource for fine mapping and positional cloning of quantitative trait loci associated with economically important traits in large yellow croaker.

## 1. Introduction

Large yellow croaker (*Larimichthys crocea* (Richardson, 1846)) is a temperate-water migratory fish that is mainly distributed in the coastal areas of East China and Southeast China [1]. It is one of the most economically important marine fish in China, with an annual cultured yield that exceeds any other net-cage-farmed marine fish species [2]. With the recent rapid development of the culture industry, a series of problems, including lower growth rate, poor disease resistance, and lower viability, have been emerging in the cultured large yellow croaker [3]. Therefore, genetic improvement for important economic traits has been considered a potentially effective means to overcome these problems [3,4]. In addition, large yellow croaker is especially sensitive to air exposure and hypoxia. This makes the large yellow croaker’s case suitable for investigating response mechanisms to environmental stress [5,6].

High-resolution genetic maps are essential for fine mapping of complex traits, genome assembly, and comparative genomic analysis. To date, linkage maps have been used to study economically important traits in over 13 fish species [7]. In the previous study, genetic linkage maps were constructed using amplified fragment length polymorphism (AFLP) and microsatellite markers in large yellow croaker and one sex-linked marker that co-segregated with an AFLP marker was mapped to the male map [4]. Recently, a genetic linkage map of large yellow croaker was constructed using type II microsatellites and expressed sequence tag (EST)-derived microsatellites in two half-sib families (two females and one male), and seven quantitative trait loci (QTLs) were identified for growth traits on five linkage groups [8]*.* However, these genetic maps of large yellow croaker can only be applied to map a limited number of QTLs for a few economic traits. Thus construction of a high-resolution genetic linkage map is necessary for fine-scale mapping of important traits such as stress resistance and growth rate in large yellow croaker.

Recently, we produced a high-quality genome assembly for large yellow croaker [9]. Genomic data showed that large yellow croaker has both innate and adaptive immune systems that are relatively complete. Transcriptomic analyses of the hypoxia-exposed large yellow croaker brain further revealed novel neuro-endocrine-immune/metabolism regulatory networks that may contribute to the prevention of cerebral inflammatory injury and the maintenance of energy balance under hypoxia in fish [9]. Restriction-site associated DNA sequencing (RAD-seq) is a powerful tool for constructing high-density genetic maps, and it has been successfully applied in various species, such as stickleback (*Gasterosteus aculeatus*) [10], rainbow trout (*Oncorhynchus Mykiss*) [11], eggplant (*Solanum melongena* L*.*) [12], barley (*Hordeum vulgare*) [13], chickpea (*Cicer arietinum* L*.*) [14], guppy (*Poecilia retitculata*) [15], groupers (*Epinephelus coioides*) [16] and Japanese flounder (*Paralichthys olivaceus*) [7]. Here, we performed a large-scale identification of genome-wide single nucleotide polymorphisms (SNPs) derived from RAD-seq of a large yellow croaker mapping population containing 2 parents and 125 offspring. Then the map was used to facilitate the anchoring and orienting of assembled genome scaffolds and for chromosomal-level comparative analysis. The immunity- and hypoxia-related genes were also mapped to the pseudo-chromosomes in order to better understand the economically important traits of large yellow croaker.

## 2. Results and Discussion

### 2.1. Results

#### 2.1.1. Sequencing and Genotyping

The Hiseq 2000 sequencing generated about 1.42 billion 90-base reads. After removing low-quality raw reads, 1.32 billion high-quality reads were obtained. The average count of RAD tags per individual was 10,409,449. RAD tags were aligned and 247,179 candidate RAD loci were identified. To explore the F1 mapping population, 31,191 SNPs were detective and were used to score the progeny. Among them, 10,150 SNPs that were consistent with Mendelian segregation pattern were retained. Then they were used for the linkage map construction. The sequence dataset about each sample is shown in Table S1.

#### 2.1.2. Construction of the Genetic Map

**Figure 1:**
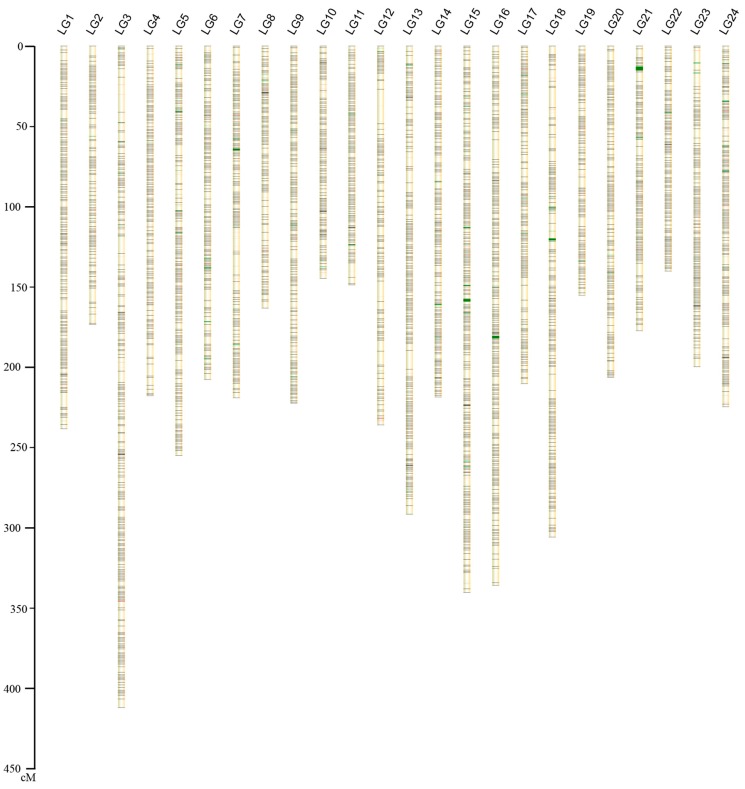
Linkage group lengths, genes involved in immunity and hypoxia adaptation, and marker distribution of the high-density SNP genetic map of large yellow croaker. NOTE: in each group, black lines represent SNPs, red lines represent genes involved in immunity and green lines represent genes involved in hypoxia adaptation. Genetic map details were given in Table S2.

Based on RAD-based sequencing of a pseudo-testcross population, the remaining 10,150 SNP markers were used to construct the first high-resolution genetic map of the large yellow croaker using Lep-MAP [17]. All putative SNPs were successfully grouped into 24 LGs (Table 1 and Figure 1), which matches with the haploid chromosome number of the large yellow croaker [18]. The total genetic distance was 5451.3 cM and the average inter-marker distance was 0.54 cM. For details, each LG contained 423 SNPs and the average length was 227.14 cM. The longest LG was LG3 with a genetic length of 412.46 cM, containing 517 SNPs. Whereas the shortest LG was LG22 with a genetic length of 140.4 cM, having 411 SNPs. Details of SNP names and positions on each LG are listed in Table S2.

**Table 1:** Characteristics of genetic maps and pseudo-chromosomes of the large yellow croaker.

Linkage Group ID	Markers Used for Genetic Map	Length (cM)	Average Inter-Loci Distance	Markers Used in Anchoring to Chromosome	Numbers of Scaffolds in Chromosome	Length (Mb)	Pseudo-Chromosome ID
LG1	504	238.74	0.47	115	33	21.58	chr1
LG2	310	173.62	0.56	67	16	10.78	chr2
LG3	517	412.46	0.80	122	23	19.38	chr3
LG4	397	218.16	0.55	78	25	23.68	chr4
LG5	467	255.29	0.55	138	35	24.60	chr5
LG6	447	207.93	0.47	90	29	17.75	chr6
LG7	471	219.35	0.47	101	41	29.75	chr7
LG8	341	163.48	0.48	99	13	10.83	chr8
LG9	409	222.76	0.55	189	29	25.66	chr9
LG10	437	144.85	0.33	56	14	7.66	chr10
LG11	380	148.97	0.39	59	7	8.85	chr11
LG12	221	236.23	1.07	142	13	13.88	chr12
LG13	634	291.87	0.46	114	24	18.96	chr13
LG14	406	218.84	0.54	52	9	6.91	chr14
LG15	645	340.75	0.53	173	54	46.00	chr15
LG16	595	336.27	0.57	99	14	12.52	chr16
LG17	355	210.61	0.59	159	19	15.55	chr17
LG18	358	306.07	0.86	117	21	11.65	chr18
LG19	302	155.54	0.52	247	16	13.46	chr19
LG20	339	206.53	0.61	105	21	13.41	chr20
LG21	347	177.56	0.51	139	13	10.30	chr21
LG22	411	140.4	0.34	167	13	9.53	chr22
LG23	461	199.99	0.43	99	25	23.34	chr23
LG24	396	225.01	0.57	162	26	25.19	chr24
Average	423	227.14	0.54	120.375	42.64	17.55	
Total	10,150	5451.3	0.54	2889	533	421.22	

#### 2.1.3. Chromosomal Assembly and Comparative Genome Analysis

**Figure 2:**
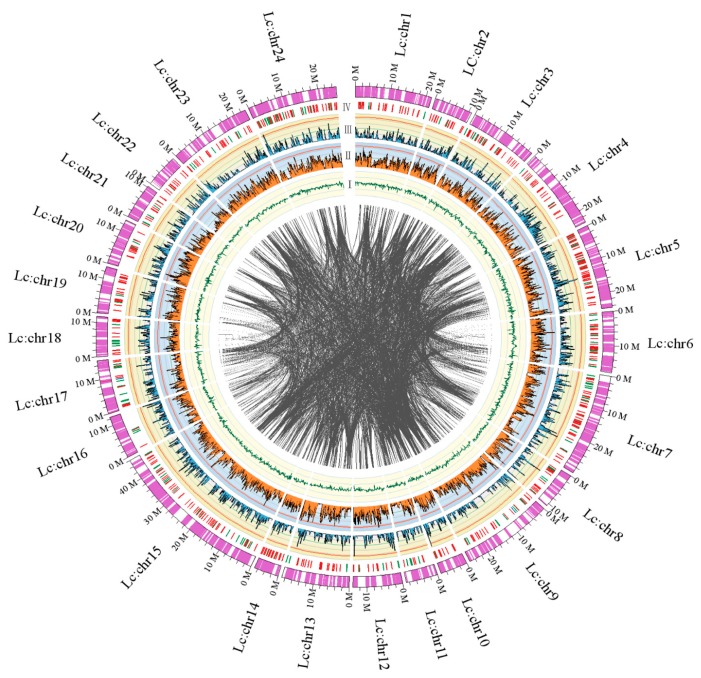
Circos atlas representation of the chromosome scaffold assembly information. The most external circle represents the length of each pseudo-chromosome. From inside to the outside ring: (**I**) GC content of 100-kb genomic interval. Each colored line represents a colinear match between two chromosomes; (**II**) Density of SNP distribution of 100-kb genomic interval; (**III**) Density of gene distribution of 100-kb genomic interval; (**IV**) Distribution of genes involved in immunity and hypoxia adaptation. The line colors of this ring were similar to those of Figure 1.

According to the genetic maps and SNPs in scaffolds, 2889 best SNPs, which covered 533 scaffolds, were chosen to assemble chromosomes using custom Perl scripts. Then, we assembled 24 pseudo-chromosomes of large yellow croaker representing 421.22 Mb, which comprised 62.04% of the genome assembly. The average chromosome length was 17.55 Mb, containing 42.64 scaffolds. The largest chromosome was chr15 with 46.00 Mb containing 173 SNPs anchored to 54 scaffolds. The smallest chromosome was chr14 with 6.91 Mb containing 52 SNPs anchored to 9 scaffolds (Figure 2).

**Figure 3:**
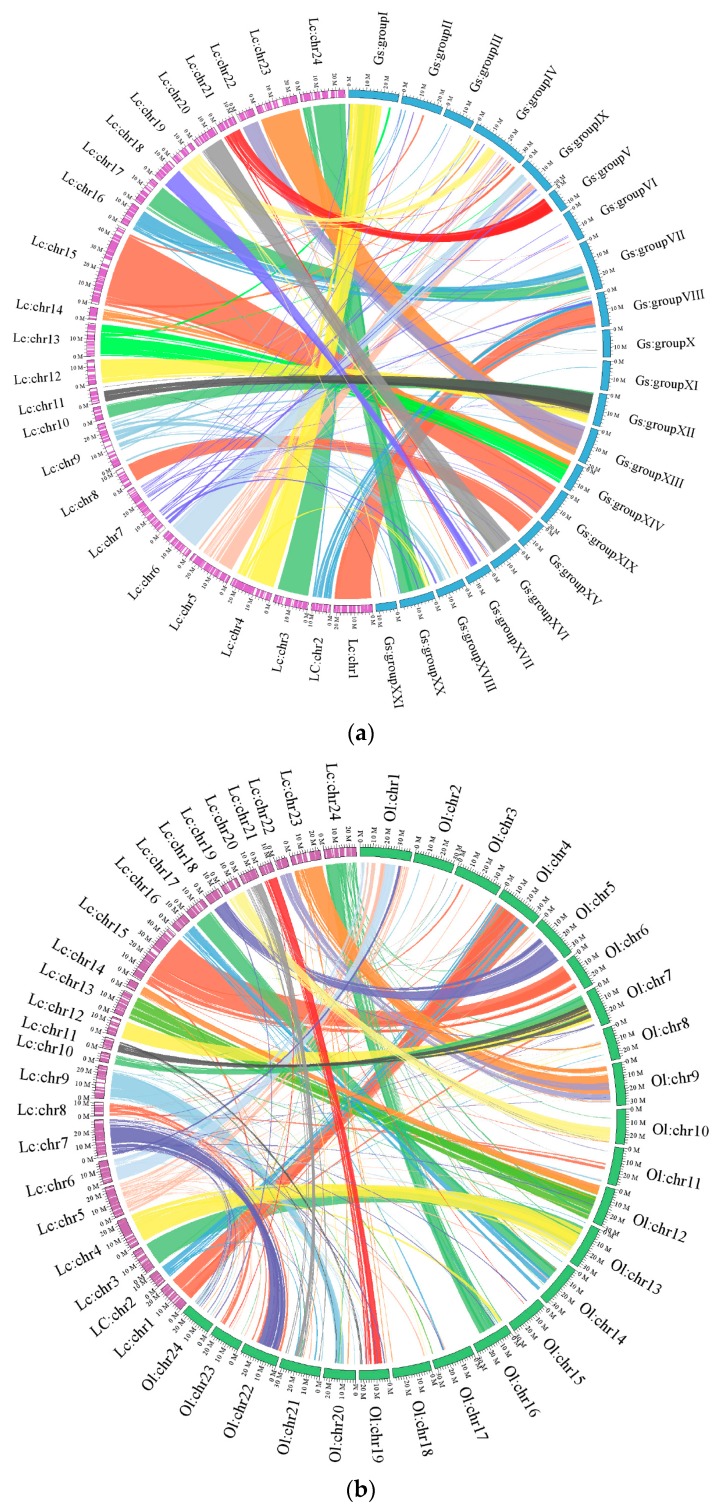
Circos atlas represents the collinear relationships between large yellow croaker and (**a**) stickleback and (**b**) medaka. The circle represents the length of each chromosome. Each colored line presents an orthologous match between two species. Ol: *Oryzias latipes*; Gs: *Gasterosteus aculeatus*; Lc; *Larimichthys crocea*.

Whole genome alignments to identify homologous synteny blocks between large yellow croaker and medaka/stickleback revealed the relation of syntenic regions among the three species. After filtering short alignment sequences (length < 100,000 bp, block larger than 100,000 bp as the synteny block), the relation between large yellow croaker and medaka/stickleback were detected (Figure 3). More specifically, there were 337 synteny blocks between large yellow croaker and medaka and the total length of these blocks was 326 Mb (account for 77% of our assembled chromosomes). Half of the 24 chromosomes of large yellow croaker (Lc: chr1, chr7, chr9, chr11, chr15, chr16, chr18, chr20, chr21, chr22, chr23 and chr24) had relatively conserved collinear blocks on medaka chromosomes (Ol: chr4, chr22, chr20, chr7, chr6, chr14, chr5, chr21, chr19, chr9 and chr16, respectively). Whereas between large yellow croaker and stickleback there were only 233 synteny blocks and the length was 352 Mb (account for 83% of assembled chromosomes). There was greater synteny and colinearity between large yellow croaker and stickleback, reflecting the closer evolutionary relationship between large yellow croaker and stickleback than that between large yellow croaker and medaka.

#### 2.1.4. Mapping of Immunity- and Hypoxia-related Genes

The annotation of immunity- and hypoxia-related genes in pseudo-chromosomes of large yellow croaker was performed with BLAST-2.2.23 program. In total, 1274 immunity-related genes were mapped to the 24 chromosomes, with an average of 53 per chromosome (Figure 1). Chr15, chr3, and chr24 had the highest counts with 117, 106 and 89 genes, respectively. The chromosomes with the lowest counts were chr22 and chr10 with 16 and 18 genes (Table S3). The innate immune system of fish, acting as the first line of defense against pathogens, is more significant for fish compared to mammals [19]. Some important innate immune molecules, such as *g-lysozyme* and *c-lysozyme* are located on chr5 and chr13, *complement c1q* and *c2* are located on Chr4, *c3* is located on Chr5, *c6* and *c7* are located on chr7, *fucolectin-4* and *-5* are located on chr3. Toll-like receptors including *tlr1*, *tlr2*, *tlr3*, *tlr5S*, *tlr5m*, *tlr8*, *tlr9*, and *tlr13* are located on chr15, chr6, chr6, chr9, chr23, chr15, chr7, and chr24, respectively. Hypoxia-related genes (195) were mapped to large yellow croaker chromosomes (Figure 1). The chromosomes containing the highest number of hypoxia-related genes were chr15 with 27 genes, and then chr24 with 22 genes. Three chromosomes contained only 2 genes: chr2, chr10, and chr22 (Table S4). Interestingly, chr15 had the highest numbers of both immunity-related genes (such as *tlr1*, *tlr8*, *nlrc3*, *nlrc5*, *nlrp12*, *trim16*, *trim25*, *irf2*, *irf7*, *il-4ra*, *il-17ra*, *il-34*, *cd9*, *cd44*, *cd59*, *cd82*, *cd151*, and *cd276)* and hypoxia-related genes (such as *hif-2*α, *amd*, *gpi*, and *wt1*).

### 2.2. Discussion

#### 2.2.1. The High-resolution Genetic Map of Large Yellow Croaker

In this study, a novel high-resolution genetic map of large yellow croaker containing 10,150 SNPs was generated, which spanned 5451.3 cM with an average inter-marker distance of 0.54 cM, representing a significant improvement over the previous linkage maps constructed by microsatellite markers [8]. A high-density linkage map can anchor *de novo* genome sequences and orient scaffolds into chromosome scale sequences [20]. Using markers to target specific scaffolds, we assigned 533 scaffolds in the large yellow croaker genome assembly to the 24 linkage groups. Approximately 421.44 Mb (62.04%) of the assembled large yellow croaker sequence was assigned to the linkage groups using 2889 SNPs. In the fugu (*Fugu rubripes*) genome, 282 Mb (71.93%) of the assembled fugu genome was placed onto 22 chromosomes (GenBank assembly accession: GCA_000180615.2). The assembled chromosome sequences of medaka (GenBank assembly accession: GCA_000313675.1) and zebrafish (GenBank assembly accession: GCA_000002035.3) were 723.44 Mb (83.17%) and 1340.43 Mb (97.72%), respectively. In Japanese eel (*Anguilla japonica*), 2672 SNP markers and 115 simple sequence repeat (SSR) markers were used to anchor 151 Mb (13%) of the assembled genome sequence [21]. In this study, the abundant SNPs were used for anchoring the scaffolds; however, the total number of anchored sequences was relatively small. Such a phenomenon might result from the following two reasons. First, the current assembled genome sequence of large yellow croaker is more fragmented. The contig N50 length is 63.11 kb in the large yellow croaker assembly [9], compared to the 1258.15 kb in the zebrafish assembly version 10. Second, for the analysis of F1 mapping population, 31,191 SNPs were obtained and could anchor 1110 scaffolds, which cover 648 M genome sequences (95.4%). However, there were 21,041 SNPs deviating from a Mendelian segregation pattern. Only 10,150 segregating SNPs were successfully grouped into 24 LGs in the construction of genetic map. Compared to the 31,191 SNPs, 10,150 SNPs were not so evenly distributed throughout the entire large yellow croaker genome. Clustered markers in areas of the same scaffold or the same position were common in this genetic map. However, 2 SNPs in one scaffold were enough to orient and order this scaffold. Redundant SNPs in the same scaffold were removed, and the remaining 2889 SNPs were used for ordering the scaffolds. In fish, clustering of SNP markers on genetic linkage maps has been observed in Atlantic salmon (*Salmo salar*) [22], channel catfish (*Ictalurus punctatus*) [23] and groupers [16]. High levels of marker clustering may be associated with the large areas of highly repetitive DNA in the teleost genome that results from whole genome duplication [16].

#### 2.2.2. Genomic Synteny Based on Chromosomal Assembly Levels

Next-generation sequencing technology has enabled the generation of draft genomes for individual species and accelerated the development of comparative analyses based on the chromosomal assembly levels [24]. Whole genome alignments to identify homologous synteny block between large yellow croaker, medaka and stickleback were performed. A synteny block is a set of contiguous genes located within the same chromosome and well conserved among different species [25]. There were 337 synteny blocks shared between large yellow croaker and medaka and the total length of these blocks was 326 Mb (77% of the assembled chromosomes). There were 233 synteny blocks shared between large yellow croaker and stickleback and the length was 352 Mb (83% of the assembled chromosomes). According to the phylogenetic tree that includes seven other sequenced teleost species [9], and was constructed using 2257 one-to-one high-quality orthologues in our previous study, large yellow croaker is more closely related to stickleback than to medaka. Therefore, both chromosomal assembly and whole genome assembly comparisons support the contention that the Sciaenidae has a close affinity to the Gasterosteiformes. Additionally, multi-syntenic correspondences were exhibited both between large yellow croaker and stickleback and between large yellow croaker and medaka, although stickleback has 21 linkage groups, whereas both large yellow croaker and medaka have 24.

#### 2.2.3. Immunity- and Hypoxia-Related Genes

Genetic linkage maps represent essential evolutionary genomics tools for commercial marine fish. They help to identify genomic regions associated with complex traits subjected to selective forces [26]. Large yellow croaker has poor disease resistance and sensitivity to hypoxia. The high-resolution chromosome map of large yellow croaker was used to analyse these important traits, and immunity-related genes (1274) and hypoxia-related genes (195) were identified in the 24 chromosomes (Tables S3 and S4). Chr15 had the highest numbers of immunity-related genes (117), such as *tlr1*, *tlr8*, *nlrc3*, *nlrc5*, *nlrp12*, *trim16*, *trim25*, *irf2*, *irf7*, *il-34*, *cd9*, *cd44*, *cd59*, and *cd151* (Table S3). In mammals, TLR8 was implicated in recognizing single-stranded RNA [27]. TRIM25 is an ubiquitin E3 ligase, which could initiate antiviral signalling through ubiquitination of the IFN-β promoter stimulator-1 (IPS-1) [28]. NLRC3 could negatively regulate the innate immune activation in response to DNA viruses, to prevent type I IFN overproduction [29]. These genes, *NLRC5*, *IRF2*, and *IRF7*, all were known to play important roles in innate antiviral immunity. The hypoxia-related genes are well-distributed on each chromosome of large yellow croaker (Table S4). The hypoxia inducible factor-1 (HIF-1) is the most important factor involved in the cellular response to hypoxia [30]. Both *hif-1*α and β were found to locate on chr8 and chr24 of large yellow croaker, respectively. Other hypoxia-related genes *phd1*, *phd3*, and *vhl* are located on chr3, chr5, and chr7, respectively. Prolyl-4-hydroxylases (PHDs), acting as molecular oxygen sensors, play important roles in regulating the stability of the transcription factor HIF by reducing the affinity of the pVHL ubiquitin E3 ligase toward the HIF proteins [31]. These data will be important for inferring the evolutionary history of these important genes and better understanding the response of large yellow croaker to hypoxia stress.

## 3. Experimental Section

### 3.1. Mapping Population and DNA Isolation

A F1 full-sib family of large yellow croaker was generated from a mariculture farm (Ningde Fufa Aquatic Breeding Co., Ltd., Ningde, China) in Ningde, Fujian, China, in March 2014. Genomic DNA was extracted from fin clips of the parents and the muscle tissues of the 125 offspring (45 days old) using the phenol-chloroform protocol [32]. DNA quality was evaluated by BGI DNA Quality Control flow [16]. All experiments were performed in accordance with the Regulations for the Administration of Affairs Concerning Experimental Animals (The State Science and Technology Commission of China, Beijing, China).

### 3.2. RAD Library Preparation and Sequencing

Genomic DNA of 127 samples (2 parents and 125 offspring) were used to build RAD library. The multiplexed shotgun sequencing strategy [10] was used to construct the library. Briefly, each sample (1 μg) was digested with 1 μL of *EcoR* I (15 U/μL, restriction enzyme cut site 5′ G^AATTC 3′) (Thermo Scientific, Walthamy, MA, USA) and last 10 min in FastDigest buffer (total volume: 30 μL) at 65 °C. Barcode adapters containing a sample-specific nucleotide code were designed, following the standard Illumina adapters designed flow. Unique barcode adapters (10 μmol) of each sample were added to reaction system. Twenty-four samples were pooled within each tube. Six pools were collected and then fragments the size of 400–600 bp were chosen. Six libraries were sequenced independently on six separate lanes of the Illumina Hiseq 2000 platform (Illumina, San Diego, CA, USA) following standard protocols.

### 3.3. SNP Discovery and Genotyping

The procedure of SNP discovery and generating genotype data for each sample was as follows. Firstly, Illumina 90-bp pair-end reads with ambiguous barcodes and aligned restriction enzyme motifs were discarded. Then remaining purified reads were classified into loci and genotyped using Soapsnp Software (Available online: http://soap.genomics.org.cn/soapsnp.html). Sequence reads from the two parents were aligned to the large yellow croaker genome assembled [9], and removed monomorphic sequences, retaining those sequences with SNPs. We filtered the SNPs under the principles described previously [7]. Sequences containing these purified SNPs were filtered to construct a reference SNP sequence. The clean reads from offspring samples were then aligned to reference SNP sequence to finally determine the genotypes of samples.

### 3.4. Genetic Map Construction

We constructed the linkage map using Lep-MAP [17], and the process was as follows. Step 1: SNPs with significant Medelian segregation distortion (Chi-square test, *p* < 0.01) were discarded, and parental SNPs were removed from the candidate SNPs. The linkage map was constructed using the remaining SNPs; Step 2: purified SNPs were assigned to the genetic linkage maps by the separate chromosomes module of Lep-MAP with a logarithm of odds (LOD) score range from 2 to 10. The SNP markers’ order of LG was then defined using the order markers module. Meanwhile, the error parameters were calculated.

### 3.5. Pseudo-Chromosomes Assembly and Genome Alignment

High-density error-corrected RAD-based SNPs were used for genome scaffold assembly. To increase the accuracy of genome assembly, we chose SNPs (at least two SNPs) of each scaffold to finish assembly using custom Perl scripts. Based on genetic distances between SNP markers, the position and orientation of each scaffold were determined. And 24 pseudo-chromosomes that corresponded to the 24 LGs were assembled. The orientation of those scaffolds with one SNP was not ordered due to the lack of enough markers. Those scaffolds were directly anchored to pseudo-chromosomes. To perform the genome synteny analyses, the medaka (*Oryzias latipes*) and stickleback (*Gasterosteus aculeatus*) genomes were downloaded from the NCBI and Ensmebl ftp databases. The Lastz program (Available online: http://www.bx.psu.edu/~rsharris/lastz/) was used to finish croaker-medaka and croaker-stickleback whole genome alignments [33]. The best homology segments were selected using custom scripts. The Circos software (http://circos.ca/) was used to display the collinear diagram [34].

### 3.6. Distributions of Genes Involving in Immunity and Hypoxia Adaptation

Genes associated with immunity and hypoxia adaptation were obtained from our early research (Table S5) [9]. We used the BLAST program (Available online: http://blast.ncbi.nlm.nih.gov/Blast.cgi?CMD=Web&PAGE_TYPE=BlastNews) to search the homologous genes in our pseudo-chromosomes. High-quality sequences were selected and placed on the Circos map.

## 4. Conclusions

A high-density genetic linkage map of 5451.3 cM was constructed. Immunity-related genes (1274) and hypoxia-related genes (195) were mapped to the 24 pseudo-chromosomes of large yellow croaker. These results provide a valuable resource for fine mapping and positional cloning of quantitative trait loci associated with economically important traits such as disease- and hypoxia-resistances.

## Supplementary Materials

The following are available online at www.mdpi.com/link; Table S1: Information about samples for completed RAD sequencing. All the statistics were produced by custom Perl scripts after finishing sequencing; Table S2: Information about the SNP linkage map of large yellow croaker. The data were produced by using the order markers module of Lep-MAP; Table S3: Detailed information about the distribution of genes involved in immunity. BLAST analysis showed that 1274 immunity-related genes were identified; Table S4: Detailed information about the distribution of genes involved in hypoxia adaptation. Hypoxia-related genes (195) were identified by BLAST program; Table S5: Homologous genes used in the BLAST analysis. Homologous genes associated with immunity and hypoxia adaptation were obtained from large yellow croaker genome. Supplementary materials can be found at http://www.mdpi.com/1422-0067/16/11/25951/s1.
